# Expression of concern: A rhodamine–quinoline based chemodosimeter capable of recognising endogenous OCl^−^ in human blood cells

**DOI:** 10.1039/d5ra90035b

**Published:** 2025-04-02

**Authors:** Shyamaprosad Goswami, Sangita Das, Krishnendu Aich, Prasanta Kumar Nandi, Kakali Ghoshal, Ching Kheng Quah, Maitree Bhattacharyya, Hoong-Kun Fun, Hatem A. Abdel-Aziz

**Affiliations:** a Department of Chemistry, Indian Institute of Engineering Science and Technology Shibpur Howrah-711103 India spgoswamical@yahoo.com +91 33 2668 2916 +91 33 2668 2961 +91 33 2668 2962 +91 33 2668 2963; b Department of Biochemistry, University of Calcutta Kolkata 700019 India bmaitree@gmail.com; c X-ray Crystallography Unit, School of Physics, Universiti Sains Malaysia 11800 USM Penang Malaysia; d Department of Pharmaceutical Chemistry, College of Pharmacy, King Saud University Riyadh 11451 Saudi Arabia hfun.c@ksu.edu.sa +966-146-76220 +966-146-77335; e Department of Applied Organic Chemistry, National Research Center Dokki Cairo 12622 Egypt

## Abstract

Expression of concern for ‘A rhodamine–quinoline based chemodosimeter capable of recognising endogenous OCl^−^ in human blood cells’ by Shyamaprosad Goswami *et al.*, *RSC Adv.*, 2014, **4**, 24881–24886, https://doi.org/10.1039/C4RA03200D.

The Royal Society of Chemistry is publishing this expression of concern in order to alert readers that concerns have been raised regarding the integrity of the fluorescence image of PBMCs in Fig. 9. The authors are not able to determine precisely how this issue arose and due to the time lapsed cannot reliably provide the raw data. An expression of concern will continue to be associated with the article unless we receive conclusive evidence regarding the reliability of the data.

Laura Fisher

26th March 2025

Executive Editor, *RSC Advances*

